# Retinal and Choriocapillaris Vascular Changes in Patients Affected by Different Clinical Phenotypes of β-Thalassemia: An Optical Coherence Tomography Angiography Study

**DOI:** 10.3390/biology10040276

**Published:** 2021-03-28

**Authors:** Gilda Cennamo, Daniela Montorio, Giuliano Mazzella, Paolo Ricchi, Silvia Costantini, Anna Spasiano, Aldo Filosa, Maria Rosaria Storino, Francesca Aquila, Fausto Tranfa, Michela Grosso

**Affiliations:** 1 Eye Clinic, Public Health Department, University of Naples “Federico II”, 80131 Naples, Italy; xgilda@hotmail.com; 2 Department of Neurosciences, Reproductive Sciences and Dentistry, University of Naples “Federico II”, 80131 Naples, Italy; da.montorio@gmail.com (D.M.); giulianomazzella@gmail.com (G.M.); 3 Rare Blood Cell Disease Unit, “Cardarelli” Hospital, 80131 Naples, Italy; pabloricchi@libero.it (P.R.); silviacostantini12@libero.it (S.C.); spasiano.anna@tiscali.it (A.S.); aldo.filosa@aocardarelli.it (A.F.); 4 CEINGE-Biotecnologie Avanzate, 80131 Naples, Italy; storino@ceinge.unina.it (M.R.S.); michela.grosso@unina.it (M.G.); 5 Department of Molecular Medicine and Medical Biotechnology, School of Medicine, University of Naples “Federico II”, 80131 Naples, Italy; francescaaquila88@gmail.com

**Keywords:** transfusion dependent thalassemia, non transfusion dependent thalassemia, thalassemia minor, optical coherence tomography angiography, vessel density, spectral domain optical coherence tomography

## Abstract

**Simple Summary:**

β-thalassemia represents a hematological disorder that determines anomalous hemolysis and ineffective erythropoiesis. The patients, undergoing regular lifelong blood transfusion, show an iron overload in the tissues that requires an iron chelation therapy. Both iron accumulation and iron-chelating agents cause ocular manifestations, such as retinal pigment epithelial (RPE) degeneration, RPE mottling, cataract, optic neuropathy and retinal venous tortuosity. In this cross-sectional study, we described the retinal and choriocapillaris microvascular changes in different clinical phenotypes of β-thalassemia that may reflect a tissue hypoxia status and oxidative damages.

**Abstract:**

In this cross-sectional study we assessed the vascular alterations in retinal and choriocapillaris perfusion in patients affected by β-thalassemia, by means of optical coherence tomography angiography (OCTA). A total of 124 eyes of 62 patients (mean age 44.74 ± 5.79 years old) affected by β-thalassemia (transfusion dependent thalassemia (TDT), non-transfusion dependent thalassemia (NTDT) and minor) were compared to 40 eyes of twenty healthy subjects. We evaluated the vessel density (VD) in superficial capillary plexus, deep capillary plexus, radial peripapillary capillary, choriocapillaris and the foveal avascular zone area. The TDT group showed a statistically significant reduction in retinal and choriocapillaris VD respect to controls and the other groups (*p* < 0.05). No statistically significant difference was found in OCTA parameters between β-thalassemia minor and controls. The NTDT group showed a significant reduction in VD in deep capillary plexus respect to controls and β-thalassemia minor. Significant negative correlations were shown in TDT group between foveal avascular zone and hemoglobin (*r* = −0.437, *p* = 0.044) and between ferritin levels and VD of choriocapillaris (*r* = −0.431, *p* = 0.038). The OCTA parameters provided a deeper understanding on retinal and choriocapillaris vascular impairment affected by tissue hypoxia levels and the oxidative stress in different clinical phenotypes of the β-thalassemia.

## 1. Introduction

Beta-thalassemia (β-thalassemia) is one of the most common genetic hemoglobinopathies characterized by a defective β globulin chain synthesis leading to hemolysis and ineffective erythropoiesis [1,2,3].

The patients with transfusion dependent thalassemia (TDT) need regular lifelong blood transfusion leading to iron overload in the tissues that requires an iron chelation therapy [4,5,6,7].

Several studies reported that both iron accumulation and iron-chelating agents determined ocular manifestations, such as retinal pigment epithelial (RPE) degeneration, RPE mottling, cataract, optic neuropathy, retinal venous tortuosity [8,9,10,11]. Moreover, the angioid streaks were reported in β-thalassemia patients hypothesizing different pathogenetic mechanisms, such as vascular obstruction affecting the choriocapillary circulation or chronic hemolysis leading to iron deposition [12].

The other forms of β-thalassemia present different clinical phenotypes. β-thalassemia minor, characterized by a presence of only a gene mutation, appears with a mild anemia not requiring blood transfusions [13] such as non-transfusion dependent thalassemia (NTDT), characterized by a different genetic compound that presents a clinical phenotype between β-thalassemia minor and TDT. It causes mild to severe hemolytic anemia and only in specific situations, such as pregnancy, surgery or serious intercurrent infection requiring a few blood transfusions [14].

β-thalassemia is a hematological disorder that determines a tissue hypoxia status and oxidative damages [15,16] that may influence the retinal and choroidal circulation.

The use of optical coherence tomography angiography (OCTA), advanced and non-invasive imaging technique, provided a quantitative evaluation of retinal and choriocapillaris vascular networks, leading to a deeper understanding of their pathological alterations in several ocular diseases [17,18].

The aim of this cross-sectional study was to investigate the retinal and choriocapillaris vascular changes in patients with different forms of β-thalassemia.

## 2. Materials and Methods

### 2.1. Subjects

A total of one hundred and twenty-four eyes of 62 patients affected by β-thalassemia were enrolled in this cross-sectional study from October 2019 to February 2020 at the Eye Clinic of the University “Federico II”, Naples in collaboration with Rare Red Blood Disease Unit of Cardarelli Hospital in Naples, Italy.

The patients were classified into 3 groups: TDT, NTDT and β thalassemia minor.

A detailed analysis of the clinical and hematological parameters was performed in all participants. Hemoglobin (g/dL) and serum ferritin (ng/mL) levels, the type and duration of chelating therapy were investigated.

All patients underwent blood transfusions approximately twice a month, and deferiprone (DFP), deferasirox (DFX) and desferal (DFO) were used as chelating agents.

Forty eyes of twenty healthy subjects without any history of intraocular surgery, vitreoretinal diseases and abnormal ophthalmic examination were included in the control group.

In order to obtain a complete ophthalmological examination, all subjects underwent the evaluation of best-corrected visual acuity (BCVA), slit-lamp biomicroscopy assessment, intraocular pressure measurement, fundus examination, spectral domain (SD)-OCT and OCTA.

Exclusion criteria included previous intraocular surgery, congenital eye disease, myopia greater than 6 diopters, history of glaucoma, retinal vascular diseases, significant lens opacities to avoid low-quality OCT and OCTA images.

The study adhered to the tenets of the Declaration of Helsinki. Written informed consent was obtained from the patients enrolled in the study. The research protocol was registered on ClinicalTrials.gov (NCT04582110).

### 2.2. Spectral Domain Optical Coherence Tomography

SD-OCT (software RTVue XR version 2017.1.0.151, Optovue Inc., Fremont, CA, USA) was used to examine the retinal nerve fiber layer (RNFL) and ganglion cell complex thickness in all patients. The optic nerve head (ONH) analysis measured the retinal nerve fiber layer thickness, calculated along a 3.45-mm diameter circle around the optic disc. The ganglion cell complex thickness was measured from the internal limiting membrane to the outer boundary of the inner plexiform layer in a 7 × 7 mm grid of the macula centered 1-mm temporal to the fovea [19]. OCTA images with a signal strength index (SSI) less than 40, and residual motion artifacts were excluded from the analysis.

### 2.3. Optical Coherence Tomography Angiography

All the subjects underwent OCTA (Optovue Angiovue System, software ReVue XR version 2017.1.0.151, Optovue Inc., Fremont, CA, USA).

Macular capillary network was evaluated in scans centered on the fovea by performing a 6 mm × 6 mm area divided, according to the ETDRS classification of diabetic retinopathy, in whole image, fovea and parafovea.

The AngioAnalytic^TM^ software automatically analyzed the vessel density (VD) in two different retinal vascular networks: superficial capillary plexus, deep capillary plexus and in the choriocapillaris, as previously described [20,21]. The VD was defined as the percentage area occupied by the microvasculature in the whole scan area and in all sections [21].

The VD of the radial peripapillary capillary plexus, analyzing the whole papillary region, inside the disc and peripapillary region with an area scan of 4.5 × 4.5 mm, was automatically calculated by the Angio Vue disc mode [22].

Angio Vue software automatically calculated the foveal avascular zone area in square millimeters over the 6 mm × 6 mm macular area in the full retinal plexus [23].

The 3D Projection Artifact Removal (PAR) algorithm, included in the software, improved the quality of OCTA images.

OCTA images with an SSI less than 60 and residual motion artifacts were excluded from the analysis.

### 2.4. Statistical Analysis

The Statistical Package for Social Sciences (Version 25 for Windows; SPSS Inc, Chicago, IL, USA) was used for statistical analysis. The differences in OCTA and SD-OCT parameters among controls and the three patients study groups were evaluated by one-way analysis of variance (ANOVA) followed by Bonferroni post hoc analysis. Pearson’s correlation was used to examine the relationships among the hematological variables and structural SD-OCT, OCTA parameters in each β-thalassemia group. A *p* value of <0.05 was considered statistically significant.

## 3. Results

A total of one hundred and twenty-four eyes of 62 patients (male 26, female 36; mean age 44.74 ± 5.79 years old) with β-thalassemia were recruited in this cross-sectional study.

The patients and controls were not significantly different in terms of age (*p* = 0.855) and sex (*p* = 0.158). BCVA was not significantly different between the controls and the three study groups *p* = 0.765) (Table 1). The hemoglobin and ferritin levels turned to be statistically increased in TDT group with respect to the other two β-thalassemia study groups (*p* < 0.001).

**Figure 1:**
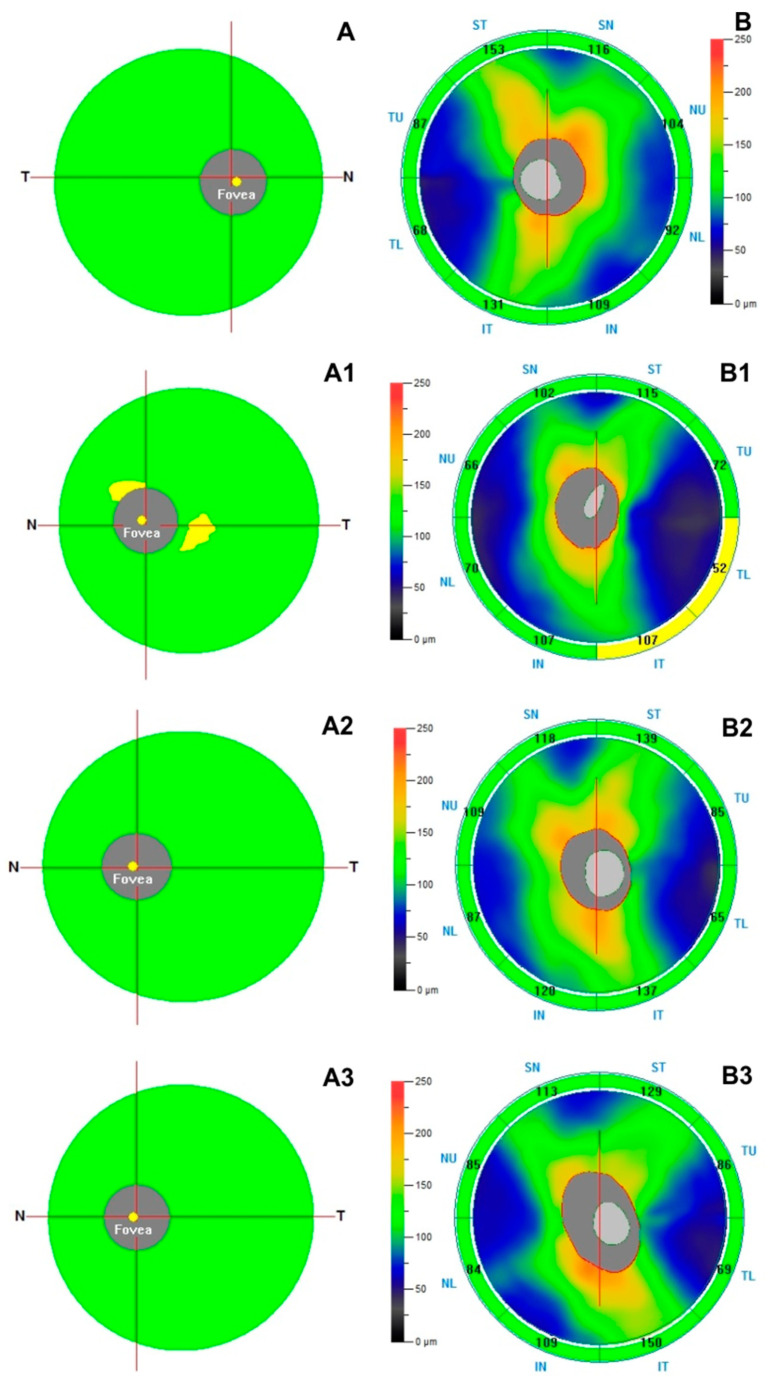
Top Row—Right eye of a 44 years-old male healthy subject shows normal thickness in Ganglion Cell Complex (GCC) (**A**) and in Retinal Nerve Fiber Layer (RNFL) (**B**) at structural spectral domain-optical coherence tomography (SD-OCT). Second Row—Left eye of a 43 years-old female patient with Transfusion Dependent Thalassemia shows focal reduction in GCC and RNFL thicknesses at SD-OCT (**A1**,**B1**). Third Row—Left eye of a 45 years-old female patient with Non-Transfusion Dependent Thalassemia shows absence of changes in GCC (**A2**) and RNFL (**B2**). Bottom Row–Left eye of a 44 years-old male patient with β-thalassemia minor shows absence of changes in GCC (**A3**) and RNFL (**B3**).

A statistically significant reduction in ganglion cell complex parameters was found in the TDT group with respect to other groups, likewise also the retinal nerve fiber layer parameters turned out to be significantly reduced with respect to controls and the study groups. No statistically significant difference was found in structural SD-OCT parameters (ganglion cell complex, retinal nerve fiber layer) between β-thalassemia minor and NTDT group with respect to controls (*p* > 0.05) (Table 2, Figure 1).

At OCTA examination, the TDT group showed a statistically significant reduction in VD of the SCP respect to controls and the other study groups in each macular sector (*p* < 0.001). The same behavior was found for VD of the deep capillary plexus, choriocapillaris, foveal avascular zone and radial peripapillary capillary plexus in the comparison between TDT and the other groups (*p* < 0.05).

The NTDT group revealed no statistically significant differences in OCTA parameters, except for VD of the deep capillary plexus that turned to be out lower respect to controls (whole image *p* = 0.034; fovea *p* = 0.043; parafovea *p* = 0.022).

**Figure 2:**
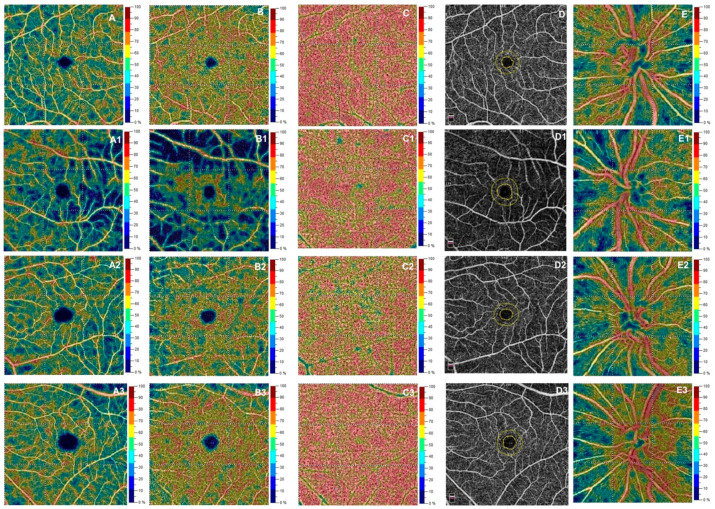
Top Row—Right eye of a 44 years-old male healthy subject reveals at OCT Angiography (OCTA) normal vessel density in superficial capillary plexus (SCP) (**A**), deep capillary plexus (DCP) (**B**), choriocapillaris (CC) (**C**), Fovea Avascular Zone (FAZ) area (**D**) and radial peripapillary capillary (RPC) (**E**). Second Row—Left eye of a 43 years-old female patient with Transfusion Dependent Thalassemia shows a reduction of vessel density in SCP (**A1**), DCP (**B1**), CC (**C1**), RPC (**E1**) and an increase of FAZ area (**D1**) respect to the healthy subjects and other groups. Third Row—Left eye of a 45 years-old female patient with Non-Transfusion Dependent Thalassemia shows absence of changes in vessel density of SCP (**A2**), CC (**C2**), FAZ area (**D2**) and RPC (**E2**) except for DCP (**B2**) that turns to be decreased respect to β-thalassemia minor and controls. Bottom Row—Left eye of a 44 years-old male patient with β-thalassemia minor shows absence of changes in vessel density of SCP (**A3**), DCP (**B3**), CC (**C3**), FAZ area (**D3**) and RPC (**E3**).

Additionally, the β-thalassemia minor showed similar values in the VD of retinal and choriocapillaris with respect to controls (*p* > 0.05) (Table 3, Figure 2).

Significant negative correlation was shown between hemoglobin and foveal avascular zone (*r* = −0.437, *p* = 0.044) and between ferritin levels and RNFL average (*r* = −0.553, *p* = 0.011), CC whole (*r* = −0.431, *p* = 0.038), choriocapillaris parafovea (*r* = −0.459, *p* = 0.041) in β-thalassemia major group. No significant correlation was found in β-thalassemia intermedia and minor (*p* > 0.05).

## 4. Discussion

To our knowledge, this is the first cross-sectional study to analyze the retinal structural and vascular changes in macular and papillary regions in patients affected by β-thalassemia in different clinical phenotypes.

In TDT patients, the evaluation of structural SD-OCT parameters revealed a significant reduction in ganglion cell complex and retinal nerve fiber layer respect to controls and other study groups.

These findings could be explained by an iron overload due to transfusion therapy in order to counteract the chronic anemia [24]. Indeed, the free iron is involved in the genesis of the retinal nerve formation and in normal functioning of the neuronal cells [25,26], but in these disorders, its high levels could cause free radicals production, due to the hemolysis, the iron chelation therapy and the decreased hepcidin levels that increase iron absorption [16,27,28,29].

According to previous studies, we hypothesized that in TDT patients, the tissue iron deposition could promote the neuronal cells death determining a thinning in ganglion cell complex and retinal nerve fiber layer, confirmed by the close correlation between the increased ferritin levels and the reduced thickness of the retinal nerve fiber layer [28,30,31].

Moreover, in our study, the patients with NTDT and β-thalassemia minor did not reveal changes in structural SD-OCT parameters, probably due to mild anemia and levels of ferritin within the normal that did not determine any neurodegenerative process, as shown also by Acer et al. [32].

The analysis of the retinal and choriocapillaris vessels by means OCTA allowed us to disclose the involvement of the microvascular networks in these disorders.

The patients with TDT showed a significant reduction in VD of the superficial capillary plexus and radial peripapillary capillary plexus with respect to other groups.

We hypothesized that both iron overload and the increased plasmatic free iron due to hemolysis, frequent blood transfusions could have determined an elevated oxidative status contributing to the retinal vascular damage in macula and papillary regions.

Moreover, the iron overload may play a key role also in the pathogenesis of endothelial dysfunction, due to the release of proinflammatory cytokines and adhesion molecules in these patients [33].

This impaired vascular perfusion may have determined a damage of the axoplasmic flow of the optic nerve, resulting in a significant retinal structural loss. Indeed, in the ganglion cell layer and the peripapillary retinal nerve fiber layer, the superficial capillary plexus and radial peripapillary capillary plexus that are responsible for their metabolic demand are localized, respectively [34].

In our results, the deep capillary plexus in TDT patients turned to be vulnerable to oxidative insult probably due to its reduced vascular caliber, smaller than superficial capillary plexus [34]. In this group, the foveal avascular zone area was involved by tissue hypoxia, showing a significant increase with respect to other β-thalassemia groups.

This area is extremely vulnerable to hypoperfusion damages that cause the drop out of the terminal interconnected capillaries at the margin of the fovea, coming from superficial and deep retinal vascular networks.

Therefore, the foveal avascular zone reflects the impairment of the retinal vessel density and it represents a sensible biomarker to ischemic insults [35]. This was confirmed by our results that showed the significant correlation between the reduced hemoglobin levels and the increased enlargement of foveal avascular zone area in TDT group.

The study conducted by Kazancı reported that in TDT, the foveal acircular index significantly correlated with hemoglobin levels, demonstrating the role of foveal avascular zone as sensible vascular biomarker in this disease [36]. Additionally, Georgalas et al. showed statistically significant differences in convexity, circularity and contour temperature of foveal avascular zone in TDT patients, with respect to controls, highlighting the relationship between the impairment of these parameters and the increased urine iron excretion [37].

In our results, the high serum ferritin levels due to hemolysis and the chronic blood transfusion significantly correlated with the choriocapillary hypoperfusion that is involved in the metabolic demand of the outer retinal layers and that could contribute to RPE damages [38,39] in TDT patients, as demonstrated by previous reports [40,41,42,43].

In this study, the patients with NTDT and β-thalassemia minor did not show changes in ganglion cell complex and retinal nerve fiber layer parameters, probably due to serum iron levels being normal.

In these groups, the retinal and the choriocapillaris microvascularization did not present significant impairment, while in NTDT, the deep capillary plexus turned to be reduced due to its reduced caliber vascular. These results could be due to the absence of a severe hypoxia and an elevated oxidative status in β-thalassemia minor and NTDT. Otherwise, the mild anemia that characterizes these disorders could have determined a vascular compensation mechanism, resulting in a recovery of the vessel density.

This supposition was confirmed by the study conducted by Acer et al., who showed that β-thalassemia minor was a wider retinal arterioral and venular caliber as a vascular response to mild hypoxia [32].

## 5. Conclusions

In conclusion, OCTA improved our knowledge of the pathophysiology of vascular perfusion in patients with β-thalassemia and could be a new vascular biomarker of this disease.

Further longitudinal studies with a wider study group and a longer follow up are needed to better explain the relationship between the retinal and choriocapillaris vascularization, structural retinal layers and the hematological parameters in different clinical phenotypes of β-thalassemia. Moreover, correlation studies between OCTA findings and the multisystem involvement in β-thalassemia patients could represent the objective of a future multidisciplinary work.

## Figures and Tables

**Table 1:** Demographic, ophthalmologic and hematologic characteristics of controls and β-thalassemia patients.

	Control Group	TDT	NTDT	β-Thalassemia Minor	*p* Value
Eye (n.)	40	42	34	48	-
Age (years)	44 ± 5.3	45.2 ± 3.6	43.88 ± 5.4	44.91 ± 7.5	0.855
Gender (male/female)	8/12	8/13	4/13	14/10	0.158 ^†^
BCVA (logMAR)	0.05 ± 0.08	0.06 ± 0.06	0.05 ± 0.06	0.03 ± 0.05	0.765
Hemoglobin (g/dL)	12.02 ± 0.90	10 ± 0.42	9.67 ± 0.86	11.58 ± 1.39	<0.001
Ferritin (nmol/L)	72.85 ± 26.36	1459.05 ± 1745.27	485.35 ± 416.89	161.52 ± 192.92	<0.001

Data are expressed as mean ± SD, TDT: Transfusion Dependent Thalassemia, NTDT: Non-Transfusion Dependent Thalassemia, BCVA: Best Corrected Visual Acuity; logMAR: logarithm of the minimum angle of resolution. One-way analysis of variance (ANOVA) followed by Bonferroni post hoc analysis. ^†^ Chi-squared test Statistical significance *p* value < 0.05.

**Table 2:** Differences in SD-OCT parameters among the controls and β-thalassemia patients.

	Control Group	TDT	*p* Value	NTDT	*p* Value *	β-Thalassemia Minor	*p* Value ^†^	Anova
GCC (µm)								
Average	103.07 ± 7.69	97.50 ± 9.23	0.011	101.35 ± 5.98	0.465	102.37 ± 8.30	0.851	0.008
Superior	105.87 ± 7.61	98.73 ± 8.74	0.001	104.17 ± 7.50	0.758	105.10 ± 9.76	0.994	0.001
Inferior	104.47 ± 7.87	96.59 ± 7.50	<0.001	100.02 ± 5.82	0.053	103.33 ± 7.15	0.742	<0.001
RNFL (µm)								
Average	110.80 ± 7.58	99.73 ± 8.48	<0.001	113.05 ± 7.24	0.819	110.25 ± 6.03	1	<0.001
Superior	112.85 ± 6.72	101.04 ± 7.47	<0.001	114.20 ± 6.79	0.784	111.20 ± 6.20	0.865	<0.001
Inferior	108.30 ± 7.75	98.47 ± 7.70	<0.001	109.29 ± 7.68	0.811	106.62 ± 6.82	0.892	<0.001

Data are expressed as mean ± SD, TDT: Transfusion Dependent Thalassemia, NTDT: Non-Transfusion Dependent Thalassemia, GCC: Ganglion Cell Complex, RNFL: Retinal Nerve Fiber Layer. *p*: TDT vs. control group, *p* *: NTDT vs. control group, *p* ^†^: β-thalassemia minor vs. control group, One-way analysis of variance (ANOVA) followed by Bonferroni post hoc analysis, Statistical significance *p* value < 0.05.

**Table 3:** Differences in OCT angiography parameters among the controls and β-thalassemia patients.

	Control Group	TDT	*p* Value	NTDT	*p* Value *	β-Thalassemia Minor	*p* Value ^†^	Anova
SCP (%)								
Whole image	50.72 ± 3.39	46.78 ± 4.37	<0.001	50.78 ± 3.51	1	52.10 ± 4.12	0.603	<0.001
Parafovea	52.56 ± 6.37	48.22 ± 6.42	0.006	53.87 ± 3.97	0.936	54.30 ± 5.66	0.999	<0.001
Fovea	23.55 ± 5.33	18.60 ± 4.03	<0.001	22.77 ± 5.25	0.945	21.20 ± 6.81	0.291	<0.001
DCP (%)								
Whole image	55.74 ± 6.31	50.40 ± 7.47	0.007	50.96 ± 7.53	0.034	55.76 ± 7.79	1	<0.001
Parafovea	58.07 ± 7.18	53.47 ± 6.24	0.022	53.58 ± 7.68	0.043	58.16 ± 7.14	1	0.001
Fovea	40.89 ± 5.82	36.55 ± 5.03	0.002	37.15 ± 5.55	0.022	40.88 ± 5.29	1	<0.001
CC (%)								
Whole image	73.78 ± 3.39	71.18 ± 4.25	0.019	72.98 ± 4.80	0.875	73.35 ± 3.30	1	0.016
Parafovea	71.73 ± 4.19	68.62 ± 4.42	0.007	70.48 ± 4.68	0.922	71.81 ± 3.90	1	0.002
Fovea	71.63 ± 5.40	68.75 ± 4.92	0.048	71.23 ± 4.59	1	72.73 ± 4.63	0.836	0.002
FAZ (mm^2^)	0.263 ± 0.08	0.320 ± 0.08	0.011	0.265 ± 0.08	1	0.261 ± 0.07	1	0.002
RPC (%)								
Whole Image	53.17 ± 4.14	46.04 ± 4.70	<0.001	51.46 ± 4.11	0.602	52.18 ± 4.64	0.958	<0.001
Inside disc	53.08 ± 5.04	49.15 ± 3.99	0.001	53.20 ± 4.25	1	53.28 ± 4.85	1	<0.001
Peripapillary region	54.74 ± 5.81	48.64 ± 4.09	<0.001	52.57 ± 4.22	0.271	53.34 ± 4.17	0.947	<0.001

Data are expressed as mean ± SD, TDT: Transfusion Dependent Thalassemia, NTDT: Non-Transfusion Dependent Thalassemia, SCP: Superficial Capillary Plexus, DCP: Deep Capillary Plexus, FAZ: Foveal Avascular Zone, RPC: Radial Peripapillary Capillary, *p*: TDT vs. control group, *p* *: NTDT vs. control group, *p* ^†^: β-thalassemia minor vs. control group, One-way analysis of variance (ANOVA) followed by Bonferroni post hoc analysis, Statistical significance *p* value < 0.05.

## Data Availability

The data presented in this study are available on request from the corresponding author. The data are not publicly available due to patients’ privacy.
